# The study of epigenetic clocks in former professional contact sports athletes with repetitive head injuries

**DOI:** 10.1002/alz70856_103202

**Published:** 2025-12-25

**Authors:** Ekaterina Rogaeva, Xuelin Tang, Vishaal Sumra, Christine Sato, Brenda Colella, Robin Green, Kaj Blennow, Henrik Zetterberg, Richard Wennberg, Charles Tator, Ming Zhang, Carmela Tartaglia

**Affiliations:** ^1^ Tanz Centre for Research in Neurodegenerative Diseases, University of Toronto, Toronto, ON, Canada; ^2^ Toronto Dementia Research Alliance (TDRA), Toronto, ON, Canada; ^3^ Clinical Center for Brain and Spinal Cord Research, School of Medicine, Tongji University, Shanghai, 200090, China; ^4^ Canadian Concussion Centre, Toronto Western Hospital, University Health Network, Toronto, ON, Canada; ^5^ Institute of Neuroscience and Physiology, Sahlgrenska Academy, University of Gothenburg, Gothenburg, Sweden; ^6^ Clinical Neurochemistry Laboratory, Sahlgrenska University Hospital, Mölndal, Sweden; ^7^ Institute of Neuroscience and Physiology, Sahlgrenska Academy at the University of Gothenburg, Göteborg, Sweden; ^8^ Hong Kong Center for Neurodegenerative Diseases, Clear Water Bay, Hong Kong; ^9^ Clinical Neurochemistry Laboratory, Sahlgrenska University Hospital, Gothenburg, Sweden; ^10^ Department of Neurodegenerative Disease, UCL Institute of Neurology, Queen Square, London, United Kingdom; ^11^ UK Dementia Research Institute at UCL, London, United Kingdom

## Abstract

**Background:**

The long‐term consequences of repetitive head impacts in contact sports include cognitive deficits, accelerated brain atrophy, and neurodegenerative diseases. Currently, no studies of former athletes have addressed the connection between brain aging and biological aging, which can be assessed using age‐related DNA methylation (DNAm) profiles. The most studied epigenetic clock is DNAm‐age, which is providing consistent results across tissues (e.g., blood and brain). It includes several measures like DNAm‐age acceleration (DNAmAA), the difference between DNAm‐age and chronological age, and DNAmAA‐residual (DNAmAAr), independent of chronological age. In a cohort of retired athletes, we explored the link between measures of brain age and epigenetic age, including the recently developed DNAmFit‐age reported to be younger in physically fit individuals.

**Method:**

We investigated 126 former contact sports athletes (mean age: 54.5±14.4; 96% male; mean concussion number: 6.8±6.7). Longitudinal assessments were available for 21 athletes (2–3 time points over 1–10 years). Bisulfite‐converted blood DNA was analyzed using the Infinium MethylationEPIC chip, and the DNAm data were submitted to the Horvath calculator (https://dnamage.clockfoundation.org/) to obtain DNAmFit‐age, DNAm‐age, and DNAmAAr. T1‐weighted MRIs were processed using the CAT12‐Toolbox. Regional gray matter volume was examined in relation to DNAmAA, DNAmAAr and DNAmFit‐age acceleration (DNAmFitAA).

**Result:**

Consistent with previous findings, chronological age was significantly associated with brain volumes and cortical thickness. DNAmAA, DNAmAAr, and DNAmFitAA remained stable over a period of up to 10 years, and none of these measures were associated with brain volumes or cortical thickness. Notably, multivariate linear regression analysis revealed a significant positive association between DNAmFitAA and the number of concussions (*p* = 0.0027, B=0.46, R^2^=0.063), indicating that every additional two concussions correspond to a 5‐year increase in DNAmFitAA.

**Conclusion:**

In former athletes, we confirmed that chronological age is associated with cerebral atrophy and identified an association between increased DNAmFitAA and a higher number of concussions. This finding requires validation in independent studies, along with an assessment of the relationship between DNAmFitAA and neurodegeneration. Our analysis did not reveal a link of the examined epigenetic clocks with brain volumes and cortical thickness. Exploration of other epigenetic clocks may provide new insights into the mechanisms underlying brain aging.

